# Treatment of Glanders

**Published:** 1901-04

**Authors:** 


### CORRESPONDENCE.

### TREATMENT OF GLANDERS.

EDITOR JOURNAL COMPARATIVE MEDICINE:

SIR: In my experimental work at the Experiment Station and Department of Veterinary Science of the North Dakota Agricultural College, I had apparently cured twenty-five cases of glanders.

All cases treated showed unmistakable evidences of the disease and reacted to the mallein test. Two of the number were inoculated with a pure culture of the bacillus malleus, one developing the chronic deep-seated form, and the other the acute superficial form of the malady. These cases yielded to the treatment quite as readily as did the others.

An autopsy on these two, and two others which were badly affected when I began the treatment, showed no lesions in the test cases, and but slight evidences of the disease in one of the others; the other one being entirely exempt from any macroscopical signs of the difficulty.

In the one case we found but a single calcareous deposit. A microscopical examination of properly prepared sections of this nodule revealed the bacilli in clumps about its margin. There were no isolated or scattering germs, giving us proof that they were in a dead or dying state.

The remainder of the animals treated were quarantined and put to ordinary farm work. They have been used in this way from two years and a half to a year and a half, according to the time the treatment was discontinued, and are still free from any symptoms of the disease. The treatment required from five to eight weeks, and costs from ten to fifteen dollars per head.

To determine the exact value of this work it would require a greater opportunity for observation and experiment than we have yet had. Owing to the fact that latent glanders may still exist in some of the subjects treated, and that the calcareous nodule found post-mortem contained the pathogenic microbe of the disease, although apparently encysted, dead or harmless, we have refrained from giving the treatment as applied.

The mistake made was that the nodule found was immediately immersed in absolute alcohol, and hence no opportunity was given to prepare a culture from it to ascertain whether life actually existed in the bacilli or not. Owing to the highly contagious and infectious nature of this disease, and the fact that man himself is subject to it, it is doubtful whether anyone would venture to take up this work and correct the errors which we have made, even though the bulletin which we have prepared was published, yet with the encouragement already obtained is there not some enthusiastic ex-station veterinarian who would like to take up and as far as possible complete this work with a view to preventing the large annual loss of horses and mules from this cause as well as to cure the disease when contracted by man himself?

No one need to undertake this work unless with ample facilities at hand under the auspices of the Government.

Sincerely yours,

W. C. Langdon, D.V.S.

Omaha, Nebraska.

---

Editor Journal of Comparative Medicine:

Dear Sir: In the March issue of the Journal your correspondent, J. P. Foster, makes a statement which is by no means justified by the facts, and which should be corrected in the interest alike of truth and of veterinary education.

He follows the too common course of stating an apparent truth in such a way as to convey an entirely false impression to the reader. Another, probably entirely innocent, example of this occurs in the same issue of the Journal, in the obituary of W. Williams. The report says: "He won the Highland Society's *first gold medal*." The facts in this case are as follows: The Highland Society gave no *gold medal*. They gave *silver medals* to the graduates who stood highest in different subjects, and one special *silver medal* to the candidate passing *the best general examination*. In 1857 they met with the difficulty that *two of the candidates were adjudged equal*, and they cut the Gordian knot by awarding *two equal silver medals*, each inscribed "*For the best general examination.*" One of these went to W. Williams and the other to James Law.

Your correspondent, J. P. Foster, affirms that graduates of the Ontario Veterinary College, who have later studied one year in the New York State Veterinary College, have on their graduation in the latter captured first prize over those more fortunate students who have had the superior (?) advantage of having passed the whole period of their professional study in the three-year school.

Now this is so stated as to be distinctly untrue. The facts are these: In 1897 two Ontario graduates were awarded the Horace K. White prizes of twenty dollars and ten dollars respectively. But the New York State Veterinary College had only opened in 1896, and had no graduates that had been three years within her walls. Again, in 1898, one Ontario graduate was awarded a Horace K. White prize, but again the New York State Veterinary College had no student that had been in her classes for three years to compete. In 1899 the students who entered the New York State Veterinary College, when it first opened its doors in 1896, were eligible for graduation, and for the first time these came into competition with graduates of the Ontario Veterinary College, who had spent one year at Cornell, and neither then nor since has any graduate of the Ontario Veterinary College been fortunate enough to carry off a Horace K. White prize.

I may add that in 1899 the University awarded a fellowship of $500 to a veterinary graduate of the year, and the choice fell on a man who had spent the whole three years of his undergraduate study in the New York State Veterinary College.

These are the plain facts, and it will be seen that they put J. P. Foster's contention entirely out of court so far as it has any bearing on this institution and its graduates.

Had it been otherwise, had the Ontario graduates *captured the prizes* in competition with the graduates of the New York State Veterinary College, it would have proved little in favor of a nominal matriculation examination, a six months', a year, and a two-years' course. The personal equation of the man is always to be considered, a man of great mental powers and assiduous devotion will rise above the limitations of his surroundings, and excel those who had far better opportunities. An Edison can dispense with a university or systematic technical school education. But Edisons are uncommon enough.

I believe, however, that all Ontario men who took prizes here had first taken prizes in Ontario. They were the selected men from a large class, and not at all the representatives of the general average. They were men likely to make their mark, go where they will. Moreover, they had each spent in the New York State Veterinary College nine months, the equivalent of three-fourths of the entire Ontario curriculum. It is, therefore, unreasonable to claim their final excellence mainly for Ontario.

It ought to be added that the veterinary prize men here in 1897 —Drs. H. R. Ryder and W. E. Howe—were respectively graduates of a normal and of a high school. Their cases, therefore, stand for the higher requirements of the New York State Veterinary College, and squarely against the lower requirements of the Ontario Veterinary College. They are the representatives of advancement and elevation as against retrogression to a low standard. To claim such men as exemplifying the sufficiency of the low standard is to claim the exact opposite of the fact.

In conclusion, it is not our purpose to unduly depreciate the Ontario Veterinary College and its graduates. Under the unfortunate limitations which it has set itself, as regards imperfect preliminary requirements, short years, and abbreviated curriculum, its teachers have worked earnestly and accomplished much. That they have not accomplished more is due to the system rather than the men, and what is wanted is a thorough revolution in the system in order to give the teachers a fair opportunity.

Very respectfully,

JAMES LAW.

CORNELL UNIVERSITY, ITHACA, N. Y., April 4, 1901.

---

EDITOR JOURNAL OF COMPARATIVE MEDICINE :

DEAR SIR : In your March number, on page 172, Dr. J. P. Foster, in writing up the Ontario Veterinary College, takes occasion to criticise certain of my statements as follows :

> "Professor Williams . . . refers to the well-known fact that students of the Ontario Veterinary College are required to pass the intervening time between their junior and senior terms with a qualified practitioner. . . . He condemns it on the theory that the veterinarian with whom the student practises may not be competent."

A careful reading of the article fails to disclose to us any serious ground for complaint. Apparently he refers to the following :

> "The veterinary college having the greatest number of graduates in America evades its duty (of teaching practical surgery) by demanding that the students shall spend their one summer vacation with private practitioners, evidently for the purpose of learning practical surgery, which would be unnecessary if it were taught in the college. The college thus unloads upon practitioners a very grave responsibility and assumes the position that while there are only two or three professors fitted to tell students in lectures what they will see in practice, any horse doctor is good enough to teach them the really practical part of the subject."

Apparently Dr. F. did not see our point. We held that a competent professor, say of clinical surgery, should with adequate material and equipment teach his subject to a student who has had but six months' study better than an ordinary practitioner could do.

We hold that in a goodly measure veterinary subjects can be taught by veterinary teachers, and that one who makes teaching his sole vocation can, for example, teach a student how to diagnose spavin with a case of spavin before him for that purpose as well as a busy practitioner can, and that the student will acquire even more knowledge by performing firing, neurectomy, or tenotomy on the patient under the teacher's directions than he would to hold a twitch or pull on a rope while the practitioner did the work.

We did not criticise the Ontario Veterinary College for what it does toward a practical education, but for what it leaves undone. That others remedy the deficiency somewhat does not invalidate the argument. Very respectfully,

W. L. WILLIAMS.

CORNELL UNIVERSITY, ITHACA, N. Y., April 4, 1901.

